# Supplementary figures and images for: Potential Role of Lauric Acid in Milk Fat Synthesis in Chinese Holstein Cows Based on Integrated Analysis of Ruminal Microbiome and Metabolome
Source: Animals (Basel). 2024 May 17;14(10):1493. doi: 10.3390/ani14101493 (PMC11117337; doi:10.3390/ani14101493)

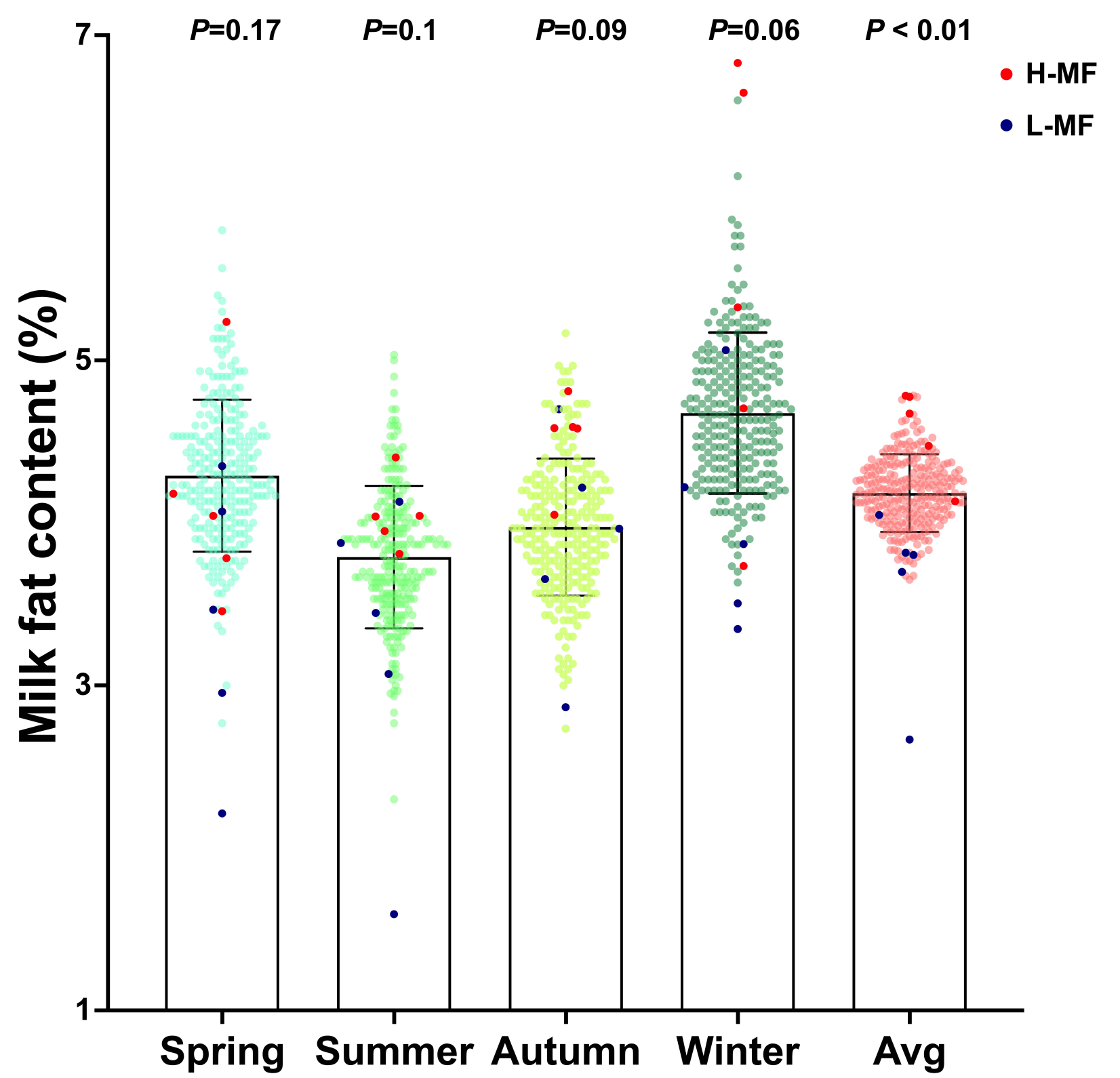

Supplement: Supplementary file 1 [file animals-14-01493-s001.zip › Figure S1.pdf]

# Top 20 of KEGG Enrichment

Pathway

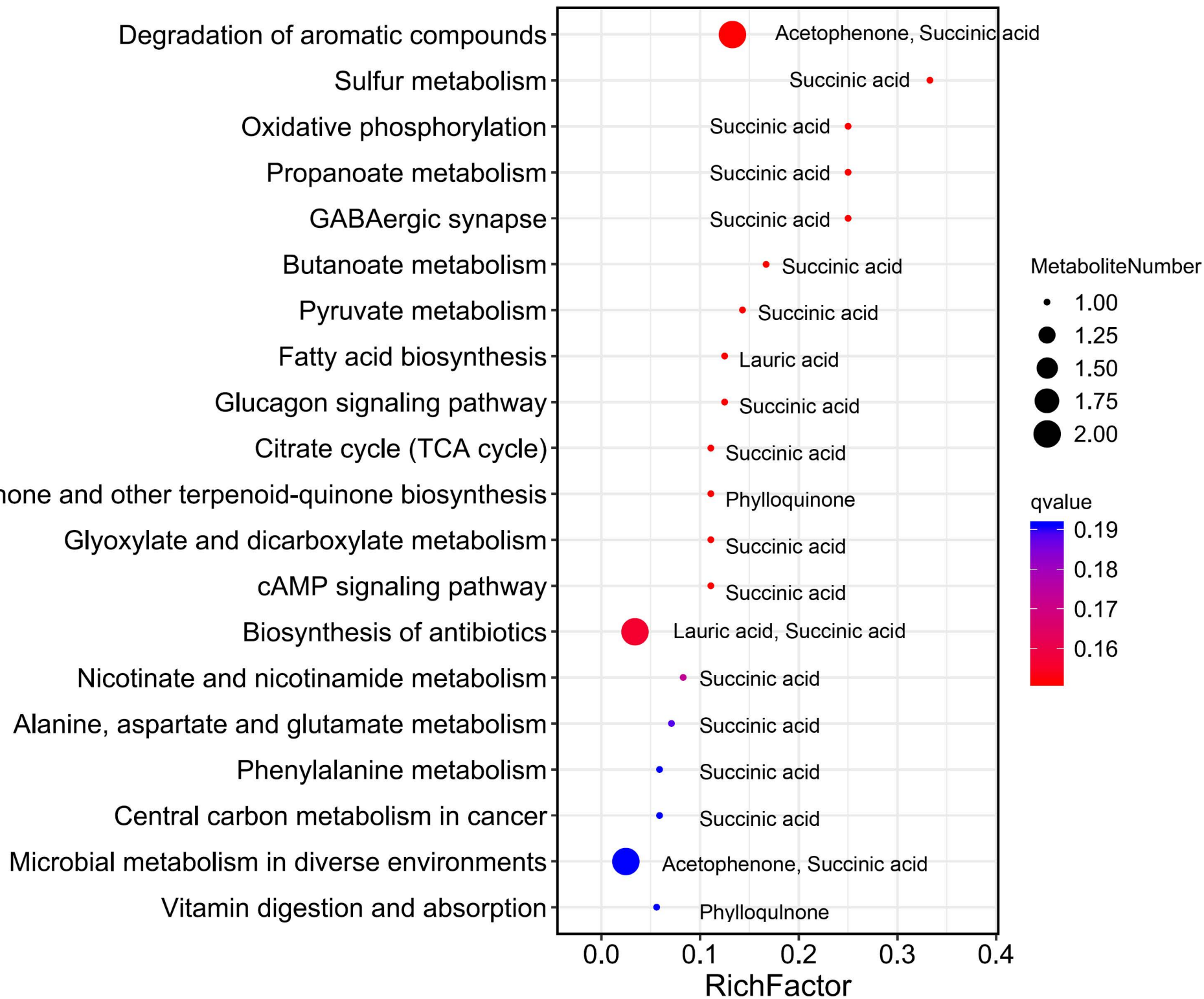

Supplement: Supplementary file 1 [file animals-14-01493-s001.zip › Figure S2.pdf]
